# Supplementary material for: Identification of translation events that drive nonsense-mediated mRNA decay reveals functional roles for noncoding RNAs
Source: bioRxiv. 2025 Aug 17:2025.08.15.670413. Preprint. [Version 1] doi: 10.1101/2025.08.15.670413 (PMC12363938; doi:10.1101/2025.08.15.670413)
Supplement: Supplement 5 [file NIHPP2025.08.15.670413V1-supplement-1.pdf]

bioRxiv preprint doi: https://doi.org/10.1101/2025.08.15.670413; this version posted August 17, 2025. The copyright holder for this preprint (which was not certified by peer review) is the author/funder. This article is a US Government work. It is not subject to copyright under 17 USC 105 and is also made available for use under a CC0 license.

SUPPLEMENTAL INFORMATION

bioRxiv preprint doi: https://doi.org/10.1101/2025.08.15.670413; this version posted August 17, 2025. The copyright holder for this preprint (which was not certified by peer review) is the author/funder. This article is a US Government work. It is not subject to copyright under 17 USC 105 and is also made available for use under a CC0 license.

Figure S1

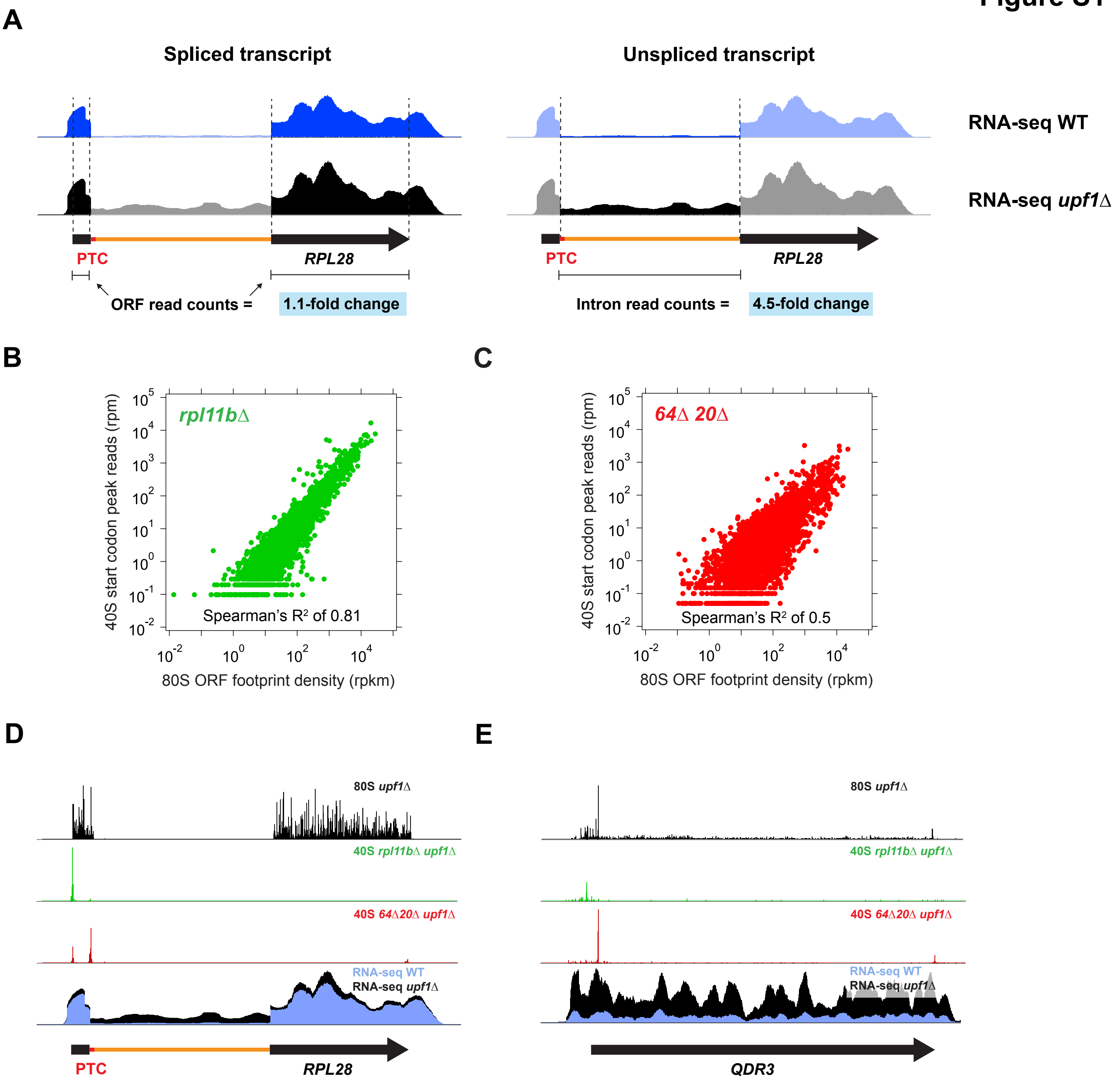

bioRxiv preprint doi: https://doi.org/10.1101/2025.08.15.670413; this version posted August 17, 2025. The copyright holder for this preprint (which was not certified by peer review) is the author/funder. This article is a US Government work. It is not subject to copyright under 17 USC 105 and is also made available for use under a CC0 license.

Figure S2

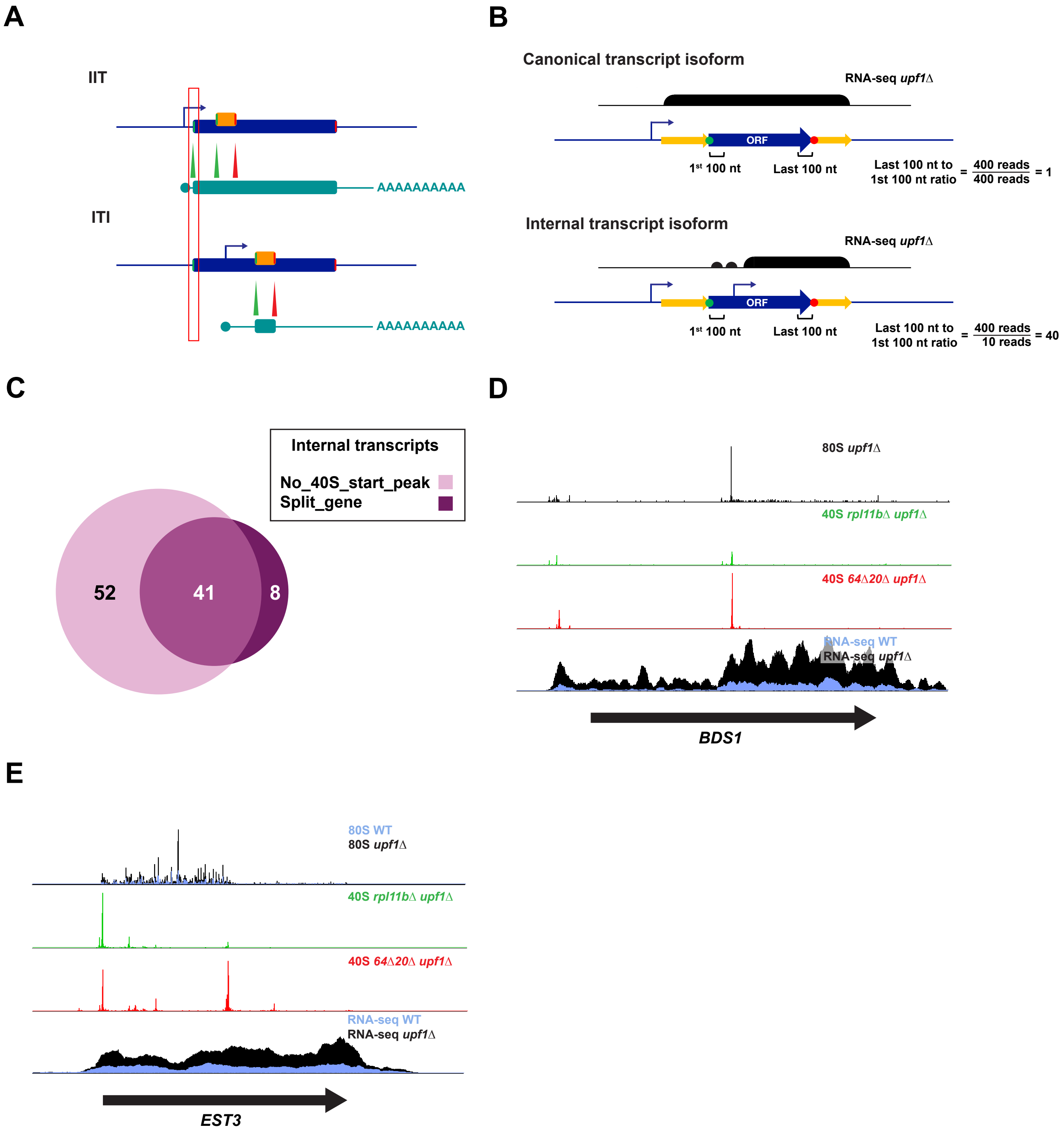

bioRxiv preprint doi: https://doi.org/10.1101/2025.08.15.670413; this version posted August 17, 2025. The copyright holder for this preprint (which was not certified by peer review) is the author/funder. This article is a US Government work. It is not subject to copyright under 17 USC 105 and is also made available for use under a CC0 license.

Figure S3

A

Bold - uORF translation detected by 40S profiling

```
AAATCCTAAAATTTATGAAAATGATTAAAAAAAGACATCCACTTCCCCATAGTGCTAAATTGGTATGGACGTTAAATAGCTTTTATCGTCGTTGGTT
TTCAGACAAATGTGTAGCACCCTAAGTGTGAGATATCATATATTTCAGAGAATATAGCACCAAGTATCGTTATGCGTTCTGGTCCGTACCTGAGAGC
TGCTCATAAGAATAGGCAAAAGCAAGAAAGAGTGTTCTGTCAGGAATTTTCAAACTATTTACAAGCTAGGAAGGCTATACGTAGTAAATAACAGCCA
CGATTCGACGTGGATAAACAAACATCTACGAAATCCCATTTTGGCATGAAAAAGTGGAGCAAGAAAGACTTGAGGTATGAAATACTTGCAATCTTTC
ACATCAACACTACTTTCAAGATGTTATTGCAAACAGAAAGTGCGATTCTCTTTCTTTCATCCGGGCGTTACAGATGACCATATCTTCAGTGAGGATA
ACGAAAGATAAAGAGTGCTGGCTCACATTAATGGAAAAAGAAATCTACCCGCTAATTGTGATGGCTCAGAGTTTCTTCTTATGATAAGGATGTTAGT
TTATATCTTGACATGTGTGTTTCTATTGTACACCGTAATAGCAGATTAGATAAACGAGAGGTCATTCGTAAGGATGAAAAAATGAACATCTGTTTAA
CTTTCAAGCTTTCTACCATATAGCCGAGATGAGCAAATACATTAAAACATCAAAAAGTGGTGCTTGAATGCTTATTTTATGACGCAAAGCAGATGTA
ATGCATCCCGAGTAAAACAGAATACTTAATTTGACTTCAGCAAAATAACTACCCAGAATGAACACCGAATTTGACATTGATAACAAGACAATCTAGG
AATACTTTTACATCAGCACAATATCCATGGTGGATCCCGGCGGATTTTTCTTTCTTATTGCACCTTAATCAGGTTTACAGCCTATGACTGACTCTGG
AGTGTCCTTTCCCAGTATCTGATTGTTTCCGCAACATCTATGCATATCAGAGAAGCGTTCTCATCAGTCACTTGACAAATGCTCGAGGAGCTATCAT
TTGCTGATAAGGTGCTACAGCGCGCTCCTGCCGCACGCTTTGTTCCTTTTCGATAAGAGTCCCTCGCGTTAGTCTGAGTGAAGTGCGGAATTCAGCA
AACGAATAACAATCGACCTTATGATCATGTGGATTATCGGGGCAAAAGATTTGGCCAAGATGTCAGAGAACGTTATCACCAATCACTCACACAATTA
AGTGGTAGTGTAACTCCGAAGATACGGCTAATACTTATCATTATCTGGTTTTCCGAATATACAGATTGGATGAAGTAATATATGTATATAAATGGAC
CAAGGAAACATCAAATTAGGAGATCATGAGGGAAAGGTTTAACATAACAACATTGAAGAAAACAACAAAACAAGGATAATCAAATAGTGTAAAAAAA
AAAATTCAAGATGTCAGCGGATGCTAGTACAAATTCGAATGCTTCCCTAGACGAAAAAAATTTAAACATCACTTCAGAAGCTGAAATCAAGAATGAA
GACGTAACCGC[illegible]GAGCCAGTTCTAAGCACGGT
```

DAL5 start codon

B

Bold - uORF translation detected by 40S profiling

```
AAATCCTAAAATTTAAAAAAAAAAATTAAAAAAAGACATCCACTTCCCCATAGTGCTAAATTGGTAAAGACGTTAAATAGCTTTTATCGTCGTTGGTT
TTCAGACAAAAAAATAGCACCCTAAGTGTGAGATATCATATATTTCAGAGAATATAGCACCAAGTATCGTTAAACGTTCTGGTCCGTACCTGAGAGC
TGCTCATAAGAATAGGCAAAAGCAAGAAAGAGTGTTCTGTCAGGAATTTTCAAACTATTTACAAGCTAGGAAGGCTATACGTAGTAAATAACAGCCA
CGATTCGACGTGGATAAACAAACATCTACGAAATCCCATTTTGGCAAAAAAAAGTGGAGCAAGAAAGACTTGAGGTAAAAAATACTTGCAATCTTTC
ACATCAACACTACTTTCAAGAAATTATTGCAAACAGAAAGTGCGATTCTCTTTCTTTCATCCGGGCGTTACAGAAAACCATATCTTCAGTGAGGATA
ACGAAAGATAAAGAGTGCTGGCTCACATTAAAAGAAAAAGAAATCTACCCGCTAATTGTGAAAGCTCAGAGTTTCTTCTTAAAATAAGGAAATTAGT
TTATATCTTGACAAAAAAATTTCTATTGTACACCGTAATAGCAGATTAGATAAACGAGAGGTCATTCGTAAGGAAAAAAAAAAAAAACATCTGTTTAA
CTTTCAAGCTTTCTACCATATAGCCGAGAAAAGCAAATACATTAAAACATCAAAAAGTGGTGCTTGAAAACTTATTTTAAAACGCAAAGCAGAAATA
AAACATCCCGAGTAAAACAGAATACTTAATTTGACTTCAGCAAAATAACTACCCAGAAAAAACACCGAATTTGACATTGATAACAAGACAATCTAGG
AATACTTTTACATCAGCACAATATCCAAAGTGGATCCCGGCGGATTTTTCTTTCTTATTGCACCTTAATCAGGTTTACAGCCTAAAACTGACTCTGG
AGTGTCCTTTCCCAGTATCTGATTGTTTCCGCAACATCTAAACATATCAGAGAAGCGTTCTCATCAGTCACTTGACAAAAACTCGAGGAGCTATCAT
TTGCTGATAAGGTGCTACAGCGCGCTCCTGCCGCACGCTTTGTTCCTTTTCGATAAGAGTCCCTCGCGTTAGTCTGAGTGAAGTGCGGAATTCAGCA
AACGAATAACAATCGACCTTAAAATCAAAAAGATTATCGGGGCAAAAGATTTGGCCAAGAAATCAGAGAACGTTATCACCAATCACTCACACAATTA
AGTGGTAGTGTAACTCCGAAGATACGGCTAATACTTATCATTATCTGGTTTTCCGAATATACAGATTGGAAAAAGTAATATAAATATATAAAAAGAC
CAAGGAAACATCAAATTAGGAGATCAAAAGGGAAAGGTTTAACATAACAACATTGAAGAAAACAACAAAACAAGGATAATCAAATAGTGTAAAAAAA
AAAATTCAAGATGTCAGCGGATGCTAGTACAAATTCGAATGCTTCCCTAGACGAAAAAAATTTAAACATCACTTCAGAAGCTGAAATCAAGAATGAA
GACGTAACCGC[illegible]GAGCCAGTTCTAAGCACGGT
```

DAL5 start codon

C

Bold - uORF translation detected by 40S profiling

```
AAGTATCACAAAGATTGGATAGTTTCGTCAATTGGTGAAGCAATAAGTATAAAATGGGGACATTTACGCATGTAGTCTTAATTCTTCACTTTCTGTG
CAGTTGATATCACTTAGAGTATGTGTCATAGGCACGGTAAAGAGCACTGAACAATGGTGAAGATAAGCTTGGACAACACTGCTCTATACGCAGACAT
CGACACGACTCCTCAATTTGAACCTTCCAAAACTACTGTAGCTGATATTTTAAC[illegible]AAAGATGCCTTAGAGTTCATTGTTTTGCTGCATAGAACT
```

DAL7 start codon

D

Bold - uORF translation detected by 40S profiling

```
AAGTATCACAAAGATTGGATAGTTTCGTCAATTGGTGAAGCAATAAGTATAAAAAAGGGACATTTACGCAAATAGTCTTAATTCTTCACTTTCTGTG
CAGTTGATATCACTTAGAGTAAAAATCATAGGCACGGTAAAGAGCACTGAACAATGGTGAAGATAAGCTTGGACAACACTGCTCTATACGCAGACAT
CGACACGACTCCTCAATTTGAACCTTCCAAAACTACTGTAGCTGATATTTTAAC[illegible]AAAGATGCCTTAGAGTTCATTGTTTTGCTGCATAGAACT
```

DAL7 start codon

bioRxiv preprint doi: https://doi.org/10.1101/2025.08.15.670413; this version posted August 17, 2025. The copyright holder for this preprint (which was not certified by peer review) is the author/funder. This article is a US Government work. It is not subject to copyright under 17 USC 105 and is also made available for use under a CC0 license.

Figure S4

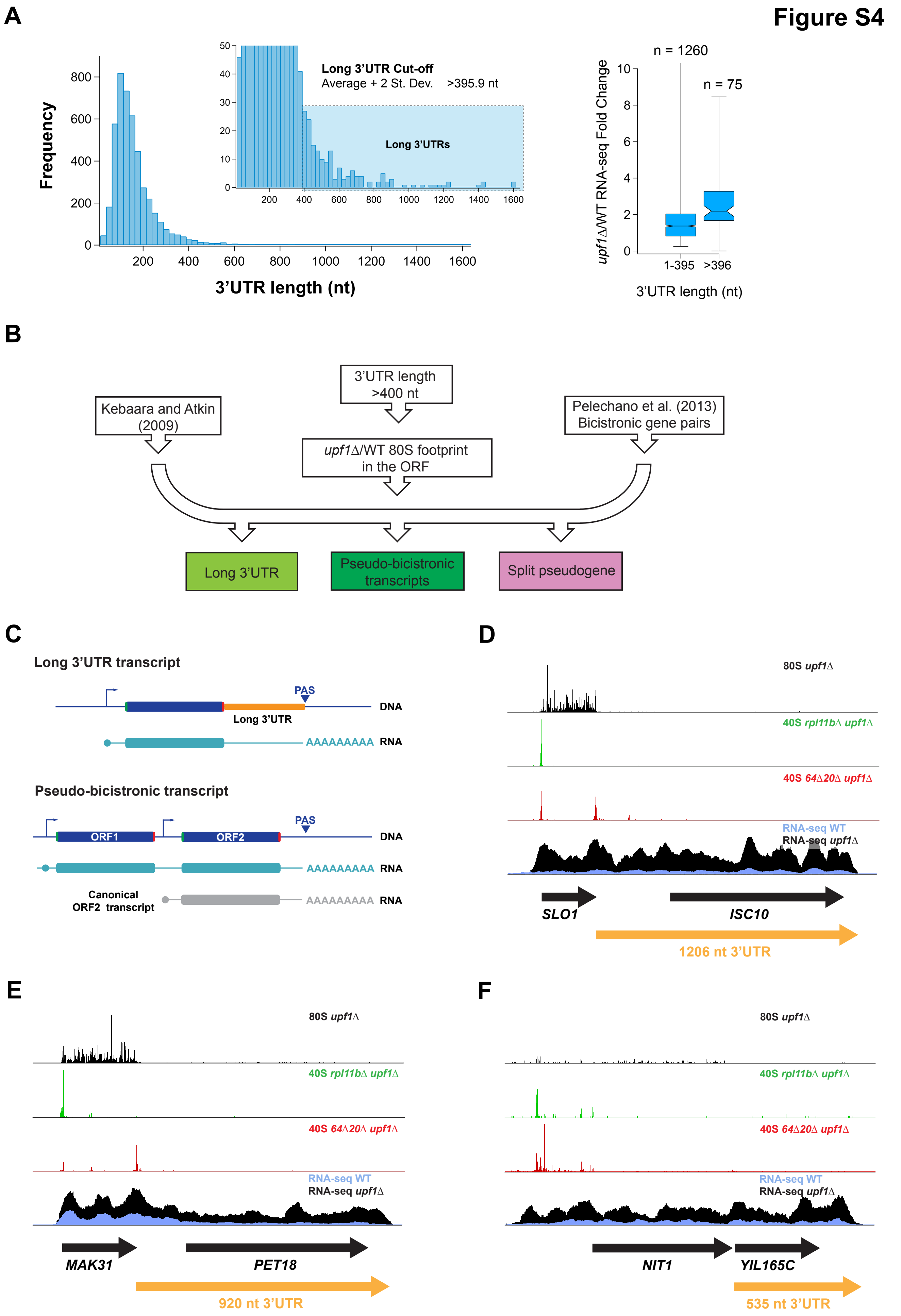

bioRxiv preprint doi: https://doi.org/10.1101/2025.08.15.670413; this version posted August 17, 2025. The copyright holder for this preprint (which was not certified by peer review) is the author/funder. This article is a US Government work. It is not subject to copyright under 17 USC 105 and is also made available for use under a CC0 license.

Figure S5

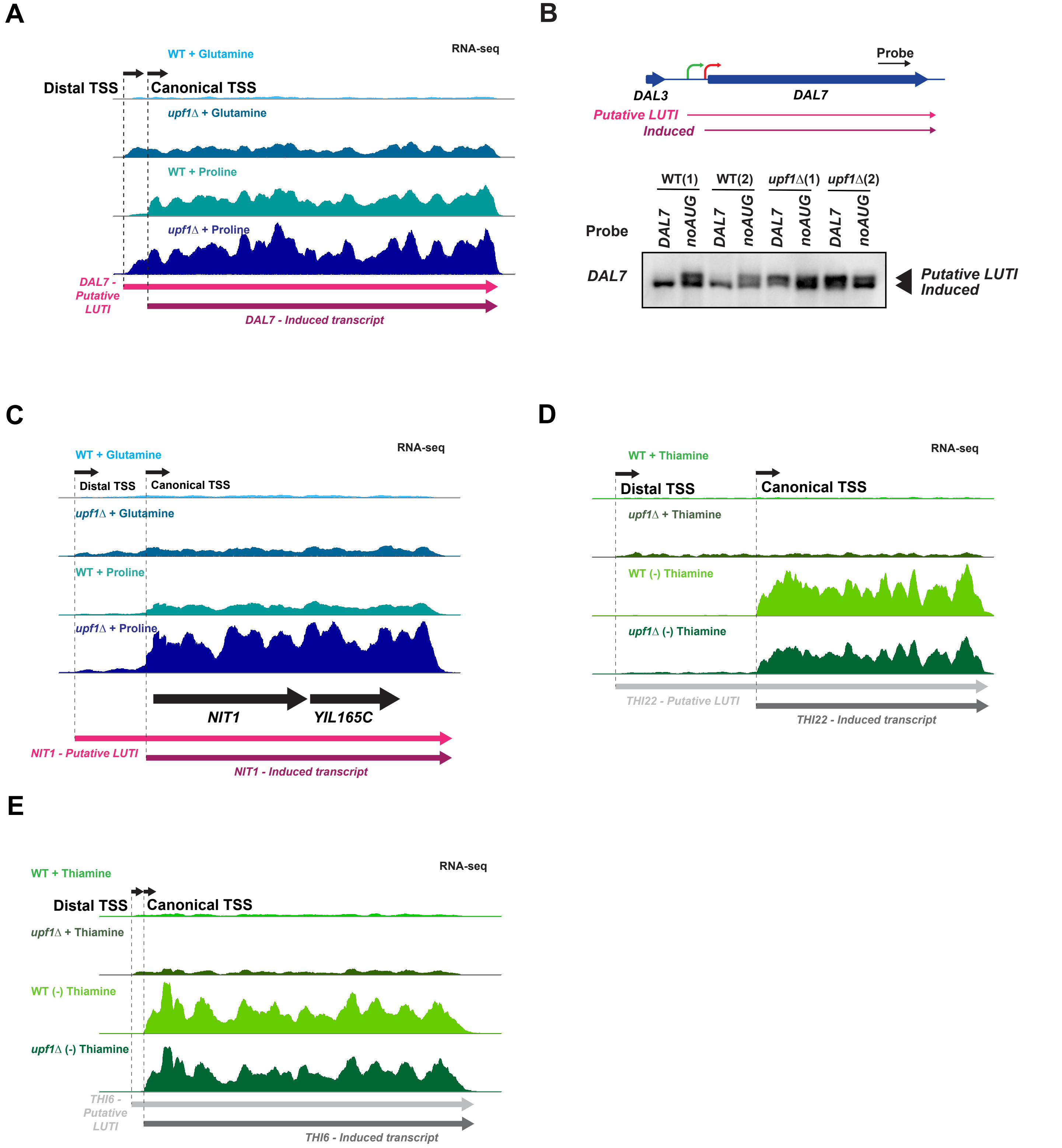

bioRxiv preprint doi: https://doi.org/10.1101/2025.08.15.670413; this version posted August 17, 2025. The copyright holder for this preprint (which was not certified by peer review) is the author/funder. This article is a US Government work. It is not subject to copyright under 17 USC 105 and is also made available for use under a CC0 license.

Figure S6

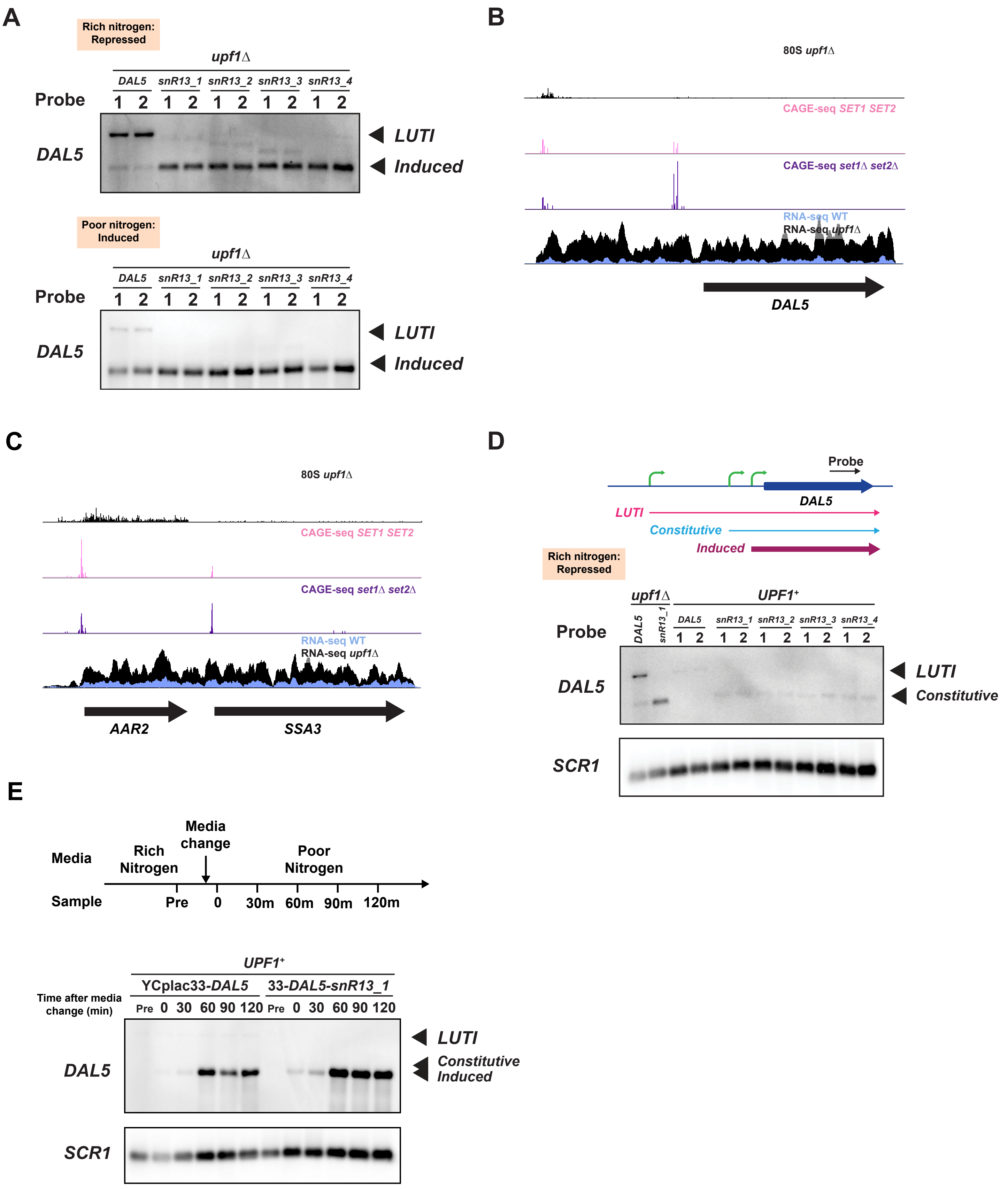

bioRxiv preprint doi: https://doi.org/10.1101/2025.08.15.670413; this version posted August 17, 2025. The copyright holder for this preprint (which was not certified by peer review) is the author/funder. This article is a US Government work. It is not subject to copyright under 17 USC 105 and is also made available for use under a CC0 license.

SUPPLEMENTAL FIGURES

Figure S1. Related to Figures 1 and 2. 40S ribosome profiling reveals translation events on NMD-sensitive transcripts.

A) mRNA-Seq data for *RPL28* demonstrating sensitivity to NMD for the intron-containing transcript but not the spliced transcript.

B) Correlation of start codon peak height in 40S profiling data from the *rpl11b*Δ strain with 80S ribosome profiling data from the *rpl11b*Δ strain demonstrates utility of using start codon peaks to locate alternative translation events. Spearman R^2 = 0.81.

C) Correlation of stop codon peak height in 40S profiling data from the *tma64*Δ *tma20*Δ strain with 80S ribosome profiling data from the *tma64*Δ *tma20*Δ strain demonstrates utility of using stop codon peaks to locate alternative translation events. Spearman R^2 = 0.50.

D) Sequencing reads for the *RPL28* gene (black arrow corresponds to main ORF exons; red thin line corresponds to the PTC; orange thin arrow corresponds to the intron) show an example of a poorly spliced transcript. 80S ribosome profiling (top) reveals translation into the intron region. 40S profiling (middle tracks) shows peaks at the main ORF start codon and the PTC in the intron and the main stop codon. mRNA-Seq (bottom) shows expression of the intron-containing transcript and its sensitivity to NMD.

E) Sequencing reads for the *QDR3* gene in rich media (arrow corresponds to main ORF) showing an example of a short-5'UTR transcript. 80S ribosome profiling (top) reveals translation of an overlapping uORF. 40S profiling (middle tracks) shows peaks at the start and stop codons of the overlapping uORF. mRNA-Seq (bottom) shows expression of the short transcript and its sensitivity to NMD.

bioRxiv preprint doi: https://doi.org/10.1101/2025.08.15.670413; this version posted August 17, 2025. The copyright holder for this preprint (which was not certified by peer review) is the author/funder. This article is a US Government work. It is not subject to copyright under 17 USC 105 and is also made available for use under a CC0 license.

Figure S2. Related to Figure 3. Analysis of translation events within the main ORFs of NMD-sensitive transcripts.

A) Differentiating between inefficient initiation transcripts (IITs) and internal transcript isoforms (ITIs). Transcripts that initiate translation inefficiently due to leaky scanning or a short transcript leader exhibit 40S peaks at the main ORF start codon in 40S profiling data whereas ITIs do not (red box).

B) Description of the split gene test used to identify ITIs. RNA-Seq reads from the first and last 100 nt of the main ORF are compared. Those genes with >2-fold more reads in the last 100 nt are considered ITIs.

C) Comparison of genes identified as ITIs using the absence of a 40S profiling peak at the main ORF start codon vs the split-gene test. Results from both tests were combined given the substantial overlap.

D) Sequencing reads for the *BDS1* gene in rich media (arrow corresponds to main ORF) showing an example of a case where both a 5'-extended transcript and ITI are expressed. 80S ribosome profiling (top) shows uORF and iORF translation. 40S profiling (middle tracks) shows locations of start and stop codons on the uORF and iORF. mRNA-Seq (bottom) shows expression of the 5'-extended transcript and ITI and their sensitivity to NMD.

E) Sequencing reads for the *EST3* gene in rich media (arrow corresponds to main ORF) showing an example of a case where a programmed frameshift leads to NMD. 80S ribosome profiling (top) shows translation up to the site of frameshift. Low frameshift efficiency results in most 80S footprints terminating just after the site of the frameshift. 40S profiling (middle tracks) shows the location of the stop codon after the frameshift site. mRNA-Seq (bottom) shows expression of the transcript and its sensitivity to NMD.

bioRxiv preprint doi: https://doi.org/10.1101/2025.08.15.670413; this version posted August 17, 2025. The copyright holder for this preprint (which was not certified by peer review) is the author/funder. This article is a US Government work. It is not subject to copyright under 17 USC 105 and is also made available for use under a CC0 license.

Figure S3. Related to Figure 4. Map of uORFs on the *DAL5* and *DAL7* transcripts.

A) Location of the 33 AUG codons in the 5'-extended transcript of *DAL5*. Translated uORFs (detected by 40S ribosome profiling) are shown in bold.

B) Location of the 33 AUG codons in the 5'-extended transcript of *DAL5* shown mutated to AAA for northern- and western-blotting experiments. Translated uORFs (detected by 40S ribosome profiling) shown in bold.

C) Same as A for *DAL7*.

D) Same as B for *DAL7*.

Underlining is used throughout to help distinguish overlapping ORFs.

Figure S4. Related to Figure 1. Analysis and assessment of genes with long 3'UTRs associated with NMD-sensitive transcripts.

A) (Left) Distribution of annotated most abundant 3'UTR lengths in yeast grown in glucose. (Right) Analysis of NMD sensitivity for transcripts with long (>396 nt) 3'UTRs vs those with short annotated 3'UTRs. On average, long 3'UTRs are more sensitive to NMD. NMD-sensitive genes included with FDR<0.01.

B) Pipeline used to determine likely cases where long 3'UTRs may sensitize transcripts to NMD and assign according to 3 output categories. Apart from the 3 inputs of gene names, the 80S footprint requirement confirms that the main ORF of the transcript targeted to NMD is translated.

C) Potential architecture of yeast genes with long 3'UTRs: a long 3'UTR is encoded for some genes (top) or a pseudo-bicistronic transcript results when the transcription terminator at the end

bioRxiv preprint doi: https://doi.org/10.1101/2025.08.15.670413; this version posted August 17, 2025. The copyright holder for this preprint (which was not certified by peer review) is the author/funder. This article is a US Government work. It is not subject to copyright under 17 USC 105 and is also made available for use under a CC0 license.

of ORF1 is either deleted or poor, leading to (at least) some production of a transcript covering both genes that acts like a transcript with long 3'UTR (bottom).

D) Sequencing reads for the *SLO1-ISC10* pseudo-bicistronic gene in rich media (black arrow corresponds to main ORFs) show this transcript is a candidate long 3'UTR transcript. 80S ribosome profiling (top) reveals translation of the first main ORF. 40S profiling (middle tracks) shows locations of start and stop codons of the first main ORF. mRNA-Seq (bottom) shows expression of pseudo-bicistronic transcript and its sensitivity to NMD. Yellow arrow indicates effective 3'UTR of *SLO1* transcript.

E) Sequencing reads for the *MAK31-PET18* pseudo-bicistronic gene in rich media (black arrow corresponds to main ORFs) show this transcript is a candidate long 3'UTR transcript. 80S ribosome profiling (top) reveals translation of the first main ORF. 40S profiling (middle tracks) shows locations of start and stop codons of the first main ORF. mRNA-Seq (bottom) shows expression of pseudo-bicistronic transcript and its sensitivity to NMD, as well as some abundance of a transcript that terminates between the two ORFs. Yellow arrow indicates effective 3'UTR of *MAK31* transcript.

F) Sequencing reads for the *NIT1-YIL165C* pseudogene transcript in rich media (black arrow corresponds to main ORFs) show this transcript is a candidate long 3'UTR transcript. 80S ribosome profiling (top) shows some translation of the first main ORF. 40S profiling (middle tracks) shows locations of start and stop codons on first main ORF. mRNA-Seq (bottom) shows expression of psuedogene transcript and its sensitivity to NMD. We note that this transcript also exhibits uORFs on an apparent 5'-extended transcript (see Figure S6A). Yellow arrow indicates effective 3'UTR of *NIT1* transcript.

bioRxiv preprint doi: https://doi.org/10.1101/2025.08.15.670413; this version posted August 17, 2025. The copyright holder for this preprint (which was not certified by peer review) is the author/funder. This article is a US Government work. It is not subject to copyright under 17 USC 105 and is also made available for use under a CC0 license.

Figure S5. Related to Figure 5. Characterization of regulated transcript pairs that include a long NMD-sensitive (putative LUTI) transcript and a downstream (canonical) transcript.

A) mRNA-Seq reads mapped to the *DAL7* gene in order from top to bottom: WT in nitrogen-rich media, *upf1*Δ in nitrogen-rich media, WT in nitrogen-poor media, *upf1*Δ in nitrogen-poor media. Black arrow on top show approximate TSSs. Colored arrows at bottom show transcripts. Data reveal that the NMD-sensitive, putative LUTI is present under both media conditions and the existence of a shorter canonical NMD-insensitive transcript is induced under poor nitrogen.

B) Northern analysis confirms expression for *DAL7* of both a putative LUTI transcript and a short canonical induced transcript under poor nitrogen conditions. The 5' uORFs confer NMD sensitivity to only the long transcript.

C) mRNA-Seq reads mapped to the *THI22* gene in order from top to bottom: WT in media with thiamine, *upf1*Δ in media with thiamine, WT in media without thiamine, *upf1*Δ in media without thiamine. Black arrow on top show approximate TSSs. Arrows at bottom show transcripts. Data reveal that the NMD-sensitive putative LUTI is present under both media conditions and the existence of a shorter canonical NMD-insensitive transcript that is induced in the absence of thiamine.

D) mRNA-Seq reads mapped to the *THI6* gene in order from top to bottom: WT in media with thiamine, *upf1*Δ in media with thiamine, WT in media without thiamine, *upf1*Δ in media without thiamine. Black arrow on top show approximate TSSs. Arrows at bottom show transcripts. Data reveal that the NMD-sensitive putative LUTI is present only in the presence of thiamine and the NMD-insensitive induced transcript is present only in the absence of thiamine.

bioRxiv preprint doi: https://doi.org/10.1101/2025.08.15.670413; this version posted August 17, 2025. The copyright holder for this preprint (which was not certified by peer review) is the author/funder. This article is a US Government work. It is not subject to copyright under 17 USC 105 and is also made available for use under a CC0 license.

E) mRNA-Seq reads mapped to the *NIT1/YIL164C-YIL165C* pseudogene in order from top to bottom: WT in nitrogen-rich media, *upf1*Δ in nitrogen-rich media, WT in nitrogen-poor media, *upf1*Δ in nitrogen-poor media. Black arrow on top show approximate TSSs. Colored arrows at bottom show transcripts. Data reveal an NMD-sensitive putative LUTI is present under both media conditions and an NMD-sensitive pseudogene transcript containing a PTC is induced under poor nitrogen.

Figure S6. Related to Figure 6. The *DAL5* LUTI represses the constitutive transcript under rich nitrogen and the induced transcript under poor nitrogen

A) Northern analysis of *DAL5* shows premature termination of LUTI transcript derepresses downstream transcripts under both rich and poor nitrogen conditions.

B) CAGE-Seq and mRNA-Seq reads mapped to the *DAL5* gene in order from top to bottom: *upf1*Δ 80S ribosome profiling (top) in nitrogen-rich media, WT (*SET1 SET2*) CAGE-Seq in nitrogen-rich media, *set1*Δ *set2*Δ CAGE-Seq in nitrogen-rich media, WT mRNA-Seq in nitrogen-rich media, and *upf1*Δ mRNA-Seq in nitrogen-rich media. Arrow corresponds to the main ORF. CAGE-Seq data reveal locations of 5'-extended (LUTI) TSS and the TSS of the constitutive transcript that is depressed in the absence of *SET1* and *SET2*.

C) CAGE-Seq and mRNA-Seq reads mapped to the *AAR2-SSA3* pseudo-bicistronic gene in order from top to bottom: *upf1*Δ 80S ribosome profiling (top) in nitrogen-rich media, WT (*SET1 SET2*) CAGE-Seq in nitrogen-rich media, *set1*Δ *set2*Δ CAGE-Seq in nitrogen-rich media, WT mRNA-Seq in nitrogen-rich media, and *upf1*Δ mRNA-Seq in nitrogen-rich media. Arrow corresponds to the main ORF. CAGE-Seq data reveals the locations of the *AAR2* TSS and the TSS of the *SSA3* transcript that is depressed in the absence of *SET1* and *SET2*.

bioRxiv preprint doi: https://doi.org/10.1101/2025.08.15.670413; this version posted August 17, 2025. The copyright holder for this preprint (which was not certified by peer review) is the author/funder. This article is a US Government work. It is not subject to copyright under 17 USC 105 and is also made available for use under a CC0 license.

D) Northern analysis of *DAL5* shows that under WT (*UPF1*$^{+}$) conditions the constitutive transcript that is derepressed upon premature termination of the LUTI transcript is NMD-sensitive.

E) Repeat of derepression experiment in 7A performed for longer timescale to establish endpoint. In blots above, 2 replicates are shown for each condition.

bioRxiv preprint doi: https://doi.org/10.1101/2025.08.15.670413; this version posted August 17, 2025. The copyright holder for this preprint (which was not certified by peer review) is the author/funder. This article is a US Government work. It is not subject to copyright under 17 USC 105 and is also made available for use under a CC0 license.

Tables S1, S2, S6, and S9 are available as downloadable files. Tables S3-S5 and S7-S8 are below.

bioRxiv preprint doi: https://doi.org/10.1101/2025.08.15.670413; this version posted August 17, 2025. The copyright holder for this preprint (which was not certified by peer review) is the author/funder. This article is a US Government work. It is not subject to copyright under 17 USC 105 and is also made available for use under a CC0 license.

Table S3: Plasmids used in this study, related to STAR Methods.

Plasmid	Description	Source
pAG32	pFA6-natMX4; *NAT* deletion cassette	Goldstein and McCusker, 1999
pFA6-kanMX4	*KAN* deletion cassette	Wach et al. 1994
YCplac33	sc[1] *URA3*	Gietz and Sugino 1988
pDY243	YCplac33-DAL5	This study
pDY236	YCplac33-DAL7	This study
pDY270	pUC57-DAL5_LUTI-noAUG	Genscript USA, Inc.
pDY284	YCplac33-DAL5_LUTI-noAUG	This study
pDY286	YCplac33-DAL7_LUTI-noAUG	This study
pDY290	YCplac33-DAL5-snR13_1	This study
pDY292	YCplac33-DAL5-snR13_2	This study
pDY294	YCplac33-DAL5-snR13_3	This study
pDY296	YCplac33-DAL5-snR13_4	This study

[1]sc, single-copy

bioRxiv preprint doi: https://doi.org/10.1101/2025.08.15.670413; this version posted August 17, 2025. The copyright holder for this preprint (which was not certified by peer review) is the author/funder. This article is a US Government work. It is not subject to copyright under 17 USC 105 and is also made available for use under a CC0 license.

Table S4: Oligonucleotides used in this study, related to STAR Methods.

Primer No.	Primer Name	Primer Sequence 5' - 3'
**Primers used for yeast strain construction**		
DYP1	kanB	CTGCAGCGAGGAGCCGTAAT
DYP2	kanC	TGATTTTGATGACGAGCGTAAT
DYP		
DYP814	UPF1-A	TTTAGTATCATCAGTTTCCCTTTGC
DYP815	UPF1-B	TGATTAAACGAGCTTTCAATTTTTC
DYP816	UPF1-C	TATGGAACCATCTGTTAATCCACTT
DYP817	UPF1-D	TTCATTAGAAGTACAATGGTAGCCC
DYP826	UPF1-A'	TGAATGCTTTTACTTTTAACTTTAG
DYP827	UPF1-D'	AATAAAAAGAACATAAGCAAAAATG
DYP1130	DAL5-MX4f	CATTCAGAGGTGGTTCATTAGTCGCCTACAGCTGCCCGTTTGGAATGACGGCCCGTAGAGGACACGTTAATAGAAGACAT**CAGCTGAAGCTTCGTACGC**
DYP1131	DAL5-MX4r	AGCAGTAACAAAATTACATAAAATACTCAATGTTGTATGTCTAATAGCGTGGTAGCCATTTTTAGTCGCCTCCCCAATCC**GCATAGGCCACTAGTGGATCTG**
DYP1136	DAL7-MX4f	AAAGTCTGGACCCTGACAGGCCCGAGAAGGACTGTGTGGAAGAACACTACAGCGATGGCGACGTTTGTATTATCATCTAA**CAGCTGAAGCTTCGTACGC**
DYP1137	DAL7-MX4r	GTCTTTTCAATGGTTTCCCGCAAGGACACCGTCATTGACTTCGAACTATTAGGATTCACAACAAGTATTCTTGTTTCCAT**GCATAGGCCACTAGTGGATCTG**
DYP1132	DAL5-A'	AAGGGAGACCTCGACGTGTGACTTG
DYP1133	DAL5-B'	ATTCTCACAATGGAAGACATATCGG
DYP1134	DAL5-C'	ATCACGCTACTACGACAAGACTCGG
DYP1135	DAL5-D'	TGTTCTTTACGATACTTTCAAGAGG
DYP1138	DAL7-A'	CGTTGAGGGCTTTTACATGTTTGGG
DYP1139	DAL7-B	CAAAACAATGAACTCTAAGGCATCT
DYP1140	DAL7-C'	TTTATTGTATGATGAAATTATTAAG
DYP1141	DAL7-D'	TGGTGGCGACGTGTCTGGCCCTGTG
DYP1178	DAL5-A''	CGAAGGTTCTATGCATCCAAAGCGG
DYP1179	DAL5-D''	ATTAATCTAATCCTTGGCCTTTGTG
DYP1180	DAL7-A''	GCGGAAGTAGCATACTTGGTAGTAG
DYP1181	DAL7-D''	CAATTAATATCCCGTTGAATTTCTG

bioRxiv preprint doi: https://doi.org/10.1101/2025.08.15.670413; this version posted August 17, 2025. The copyright holder for this preprint (which was not certified by peer review) is the author/funder. This article is a US Government work. It is not subject to copyright under 17 USC 105 and is also made available for use under a CC0 license.

Primer No.	Primer Name	Primer Sequence 5' - 3'
**Primers used for plasmid construction**		
DYP1055	YCplac33-SphI_KpnI_fwd	tataactggaGGTACCGAGCTCGAATTCAC
DYP1056	YCplac33-SphI_KpnI_rev	atcgcggggaGCATGCAAGCTTGGCGTAATC
DYP1057	DAL5_fwd	gcttgcatgcTCCCCGCGATTCGACGAG
DYP1091	DAL5_1_rev	accagataatGATAAGTATTAGCCGTATCTTCGGAGTTAC
DYP1092	DAL5_2_fwd	aatacttatcATTATCTGGTTTTCCGAATATAC
DYP1093	DAL5_2_rev	gctcggtaccTCCAGTTATATTTGCTTAAATTG
DYP34	M13r	GGAAACAGCTATGACCATG
DYP1059	DAL5seq1f	CGAGTACAAAAGGCCAGAGACAGC
DYP1060	DAL5seq2f	GTTCTGGTCCGTACCTGAGAGCTG
DYP1061	DAL5seq3f	CCTTAATCAGGTTTACAGCCTATG
DYP1062	DAL5seq4f	AAGCACGGTACTATCACCCAACGG
DYP1063	DAL5seq5f	AAGCAAGGTTTTTATCTAAAAGAG
DYP1064	DAL5seq6f	ATTTAAGGGAAAACAAGAGACGTG
DYP1065	DAL7-YXpf	TTTCAGGAGCTCTTCTGCGACTGTGGGCGAAGTAAA
DYP1066	DAL7-YXpr	TTTCAGGGATCCCGTTCGATAATTTGCTTAGCTAG
DYP1067	DAL7seq1f	TAGTCAACGCTTTGAATGCTGAAG
DYP1068	DAL7seq2f	TAAAAGATGATCCAAAGGCTAATG
DYP1257	DAL5-snR13_1_SDMf	ccttcttacattgtatcgtagcgcggatccAAAAGCAAGAAAGAGTGTTC
DYP1258	DAL5-snR13_1_SDMr	attactaagattttctacggggaagttaaaGCCTATTCTTATGAGCAG
DYP1259	DAL5-snR13_2_SDMf	ccttcttacattgtatcgtagcgcggatccATGACCATATCTTCAGTGAGG
DYP1260	DAL5-snR13_2_SDMr	attactaagattttctacggggaagttaaaCTGTAACGCCCGGATGAA
DYP1261	DAL5-snR13_3_SDMf	ccttcttacattgtatcgtagcgcggatccGCAAATACATTAAAACATCAAAAAG
DYP1262	DAL5-snR13_3_SDMr	attactaagattttctacggggaagttaaaTCATCTCGGCTATATGGTAG
DYP1263	DAL5-snR13_4_SDMf	ccttcttacattgtatcgtagcgcggatccTGACTCTGGAGTGTCCTTTC
DYP1264	DAL5-snR13_4_SDMr	attactaagattttctacggggaagttaaaGTCATAGGCTGTAAACCTG
**Primers used for constructing northern blot probe templates**		
DYP1194	DAL5_500nt_f	ATGCAGCCAATAAGAAGATACCATTTTGGAAATATAAGTTG
DYP1195	DAL5_probe_r	**TAATACGACTCACTATAGGG**TAAAGTGTATCTGAAATTTGGATTT
DYP1197	DAL7_500nt_f	ATGAAGTTTTCAGTAACATGGGTAC

bioRxiv preprint doi: https://doi.org/10.1101/2025.08.15.670413; this version posted August 17, 2025. The copyright holder for this preprint (which was not certified by peer review) is the author/funder. This article is a US Government work. It is not subject to copyright under 17 USC 105 and is also made available for use under a CC0 license.

Primer No.	Primer Name	Primer Sequence 5' - 3'
DYP1198	DAL7_probe_r	**TAATACGACTCACTATAGGG**TAATTTACTTAAGTCAACTGGTTTG
DYP1206	SCR1_500nt_f	TTTCTGGTGGGATGGGATACGTTGA
DYP1207	SCR1_probe_r	**TAATACGACTCACTATAGGG**GTTCAGGACACACTCCATCCCCGAG

bioRxiv preprint doi: https://doi.org/10.1101/2025.08.15.670413; this version posted August 17, 2025. The copyright holder for this preprint (which was not certified by peer review) is the author/funder. This article is a US Government work. It is not subject to copyright under 17 USC 105 and is also made available for use under a CC0 license.

Table S5: Yeast Strains used in this study, related to Methods.

Yeast Name	Genotype	Source
BY4741	*MATa his3Δ1 leu2Δ0 met15Δ0 ura3Δ0*	Horizon Discovery
6214	*MATa his3Δ1 leu2Δ0 met15Δ0 ura3Δ0 nam7Δ::kanMX4*	Horizon Discovery
4715	*MATa his3Δ1 leu2Δ0 met15Δ0 ura3Δ0 rpl11bΔ::kanMX4*	Horizon Discovery
YDY10	*MATa his3Δ1 leu2Δ0 met15Δ0 ura3Δ0 tma64Δ::hygMX4 tma20Δ::kanMX4*	Young et al. 2018
YDY643	*MATa his3Δ1 leu2Δ0 met15Δ0 ura3Δ0 nam7Δ::natMX4*	This study
YDY647	*MATa his3Δ1 leu2Δ0 met15Δ0 ura3Δ0 rpl11bΔ::kanMX4 nam7Δ::natMX4*	This study
YDY649	*MATa his3Δ1 leu2Δ0 met15Δ0 ura3Δ0 tma64Δ::hygMX4 tma20Δ::kanMX4 nam7Δ::natMX4*	This study
YDY677	*MATa his3Δ1 leu2Δ0 met15Δ0 ura3Δ0 dal5_extΔ::kanMX4*	This study
YDY679	*MATa his3Δ1 leu2Δ0 met15Δ0 ura3Δ0 dal7_extΔ::kanMX4*	This study
YDY691	*MATa his3Δ1 leu2Δ0 met15Δ0 ura3Δ0 nam7Δ::natMX4 dal5_extΔ::kanMX4*	This study
YDY693	*MATa his3Δ1 leu2Δ0 met15Δ0 ura3Δ0 nam7Δ::natMX4 dal7_extΔ::kanMX4*	This study

bioRxiv preprint doi: https://doi.org/10.1101/2025.08.15.670413; this version posted August 17, 2025. The copyright holder for this preprint (which was not certified by peer review) is the author/funder. This article is a US Government work. It is not subject to copyright under 17 USC 105 and is also made available for use under a CC0 license.

Table S7: 40S ribosome profiling datasets from previous papers, related to Figure S1 and Methods

Sample name	Description	Reference	GEO Number	Number mapped reads
DY135Ffx183Ffx	*rpl11b*Δ (2 rep's pooled)	Young et al. (2021)	GSM4339086	6,568,093
DY136Ffx184Ffx	*tma64*Δ/*tma20*Δ (2 rep's pooled)	Young et al. (2021)	GSM4339087	12,482,481

bioRxiv preprint doi: https://doi.org/10.1101/2025.08.15.670413; this version posted August 17, 2025. The copyright holder for this preprint (which was not certified by peer review) is the author/funder. This article is a US Government work. It is not subject to copyright under 17 USC 105 and is also made available for use under a CC0 license.

Table S8: Oligonucleotides used for ribosome profiling, related to Methods.

Primer Name	Oligonucleotide Sequence 5' - 3'
**RNA Size Marker**	
15mer	rArUrGrUrArCrArCrGrGrArGrUrCrG
25mer	rArUrGrUrArCrArCrGrGrArGrUrCrGrArGrCrArCrCrCrGrCrA
34mer	rArUrGrUrArCrArCrGrGrArGrUrCrGrArGrCrArCrCrCrGrCrArArCrGrCrGrArArUrG
50mer	rArUrGrUrArCrArCrGrGrArGrUrCrGrArCrCrCrGrCrArArCrGrCrGrArUrGrUrArCrArCrGrGrArGrUrCrGrArCrCrCrGrCrArA
70mer	rArUrGrUrArCrArCrGrGrArGrUrCrGrArCrCrCrGrCrArArCrGrCrGrArUrGrUrArCrArCrGrGrArGrUrCrGrArCrCrCrGrCrArArCrGrCrGrArUrGrUrArCrArCrGrGrArGrUrCrGrA
80mer	rArUrGrUrArCrArCrGrGrArGrUrCrGrArCrCrCrGrCrArArCrGrCrGrArUrGrUrArCrArCrGrGrArGrUrCrGrArCrCrCrGrCrArArCrGrCrGrArUrGrUrArCrArCrGrGrArGrUrCrGrArCrCrCrGrCrArArCrGrC
**Linker Oligonucleotides**	
NI-810	5´-/5Phos/NNNNNATCGTAGATCGGAAGAGCACACGTCTGAA/3ddC/
NI-811	5´-/5Phos/NNNNNAGCTAAGATCGGAAGAGCACACGTCTGAA/3ddC/
NI-812	5´-/5Phos/NNNNNCGTAAAGATCGGAAGAGCACACGTCTGAA/3ddC/
NI-813	5´-/5Phos/NNNNNCTAGAAGATCGGAAGAGCACACGTCTGAA/3ddC/
**RT Primer**	
NI-802	5´-/5Phos/NNAGATCGGAAGAGCGTCGTGTAGGGAAAGAG/iSp18/GTGACTGGAGTTCAGACGTGTGCTC
**PCR Primers**	
NI-NI-798	5´- AATGATACGGCGACCACCGAGATCTACACTCTTTCCCTACACGACGCTC
NI-799	5´-CAAGCAGAAGACGGCATACGAGATCGTGATGTGACTGGAGTTCAGACGTGTG
NI-822	5´-CAAGCAGAAGACGGCATACGAGATACATCGGTGACTGGAGTTCAGACGTGTG
NI-823	5´-CAAGCAGAAGACGGCATACGAGATGCCTAAGTGACTGGAGTTCAGACGTGTG
NI-824	5´-CAAGCAGAAGACGGCATACGAGATTGGTCAGTGACTGGAGTTCAGACGTGTG
NI-825	5´-CAAGCAGAAGACGGCATACGAGATCACTGTGTGACTGGAGTTCAGACGTGTG
NI-826	5´-CAAGCAGAAGACGGCATACGAGATATTGGCGTGACTGGAGTTCAGACGTGTG

bioRxiv preprint doi: https://doi.org/10.1101/2025.08.15.670413; this version posted August 17, 2025. The copyright holder for this preprint (which was not certified by peer review) is the author/funder. This article is a US Government work. It is not subject to copyright under 17 USC 105 and is also made available for use under a CC0 license.

Primer Name	Oligonucleotide Sequence 5' - 3'
**Subtraction Oligonucleotides**	
1b	/5BioTinTEG/GGTGCACAATCGACCGATC
2b	/5BioTinTEG/GTTTCTTTACTTATTCAATGAAGCGG
3b	/5BioTinTEG/TATAGATGGATACGAATAAGGCGTC
4	/5BioTinTEG/TTGTGGCGTCGCTGAACCATAG
5	/5BioTinTEG/CAGGGGGCATGCCTGTTTGAGCGTCAT
6	/5BioTinTEG/CGGTGCCCGAGTTGTAATTT
